# Mapping the MicroRNA Landscape of Cadmium Exposure: A Bibliometric and Bioinformatics Analysis of Molecular Targets and Regulatory Pathways

**DOI:** 10.3390/ijms27188051

**Published:** 2026-09-10

**Authors:** Muhammet Kesin, Gözde Oztan, Halim Işsever

**Affiliations:** 1 Safranbolu State Hospital, Safranbolu, Karabük 78600, Türkiye; 2 Department of Medical Biology, Istanbul Faculty of Medicine, Istanbul University, Istanbul 34093, Türkiye; gozdeoztan@istanbul.edu.tr; 3 Department of Public Health, Istanbul Faculty of Medicine, Istanbul University, Istanbul 34093, Türkiye; hissever@istanbul.edu.tr

**Keywords:** cadmium exposure, microRNA, epigenetics, bibliometric analysis, bioinformatics, oxidative stress, inflammation, apoptosis

## Abstract

Cadmium is a persistent toxic metal that promotes oxidative stress, inflammation, and epigenetic dysregulation; however, the research landscape and recurrent microRNA (miRNA)-mediated responses associated with cadmium exposure remain incompletely characterized. This study integrated bibliometric, structured content, and bioinformatics analyses to identify major research trends and recurrent miRNA signals. PubMed/MEDLINE, Scopus, and Web of Science Core Collection were searched using predefined cadmium- and miRNA-related search terms, yielding 1511 source records. After language/document-type filtering and cross-database deduplication, 817 unique records remained for screening, of which 286 publications formed the final bibliometric corpus. Author-keyword co-occurrence analysis was performed using a minimum occurrence threshold of ≥3, with ≥5 used as a sensitivity analysis. Cadmium and microRNA remained the dominant thematic core, while heavy-metal toxicity, apoptosis, and cadmium-stress responses represented the most recurrent secondary themes. In the final human-relevant biological evidence corpus (*n* = 32 studies), recurrent miRNAs showed substantial directional heterogeneity rather than a uniformly replicated response: miR-155 was reported in four studies with an equal number of increases and decreases, whereas the let-7 and miR-30 families showed modest downward tendencies, and miR-146b and miR-222 showed modest upward tendencies. These small study counts preclude strong claims of directional reproducibility. The miRNA–target network component should therefore be interpreted as exploratory and hypothesis-generating rather than as evidence of cadmium-specific pathway convergence. Overall, this synthesis supports a broader and more reproducible literature map while emphasizing the need for standardized exposure assessment, miRNA measurement, and independent validation of candidate regulatory signals.

## 1. Introduction

### 1.1. Public Health Significance of Cadmium

In general, the term “heavy metals” refers to elements with a density greater than 5 g/cm^3^ [1]. Cadmium (Cd) is a typical heavy metal characterized by high toxicity and carcinogenicity, considerable mobility, and strong chemical reactivity [2]. Cd has been identified by the World Health Organization (WHO) as one of the four major heavy metals of significant public health concern [3]. Environmental pollution is regarded as one of the most important public health problems of the twenty-first century. According to estimates based on Global Burden of Disease (GBD) 2019 data, pollution caused approximately 9 million deaths in 2019 and was responsible for one in every six deaths worldwide [4]. Cd, identified by the WHO as one of the ten chemicals of major public health concern, poses substantial risks to human health [5].

Cd, a toxic element with no essential biological function in humans, is released into the environment through various routes, including fossil fuel combustion, mining activities, the application of sewage sludge and fertilizers, and industrial waste discharge. Human exposure to Cd occurs through ingestion, inhalation, and dermal absorption; dietary intake is the primary route among individuals without occupational exposure and among nonsmokers [6].

According to JECFA, the biological half-life of cadmium in the human body ranges from approximately 16 to 30 years [7]. Cd reportedly undergoes long-term bioaccumulation in the human body, reaching steady-state levels in the kidneys only after 45–60 years of exposure [8]. Therefore, even chronic low-level exposure can lead to significant health effects over time. Certain pulmonary diseases, such as emphysema, asthma, and bronchitis, as well as hypertension, are thought to be associated with chronic poisoning. The manifestations of cadmium poisoning may vary according to the duration of exposure, dietary patterns, and the age and health status of exposed individuals. For nonsmokers and individuals without occupational exposure, diet is the primary source of exposure [9].

Heavy-metal contamination of food is a serious global problem, particularly in polluted agricultural regions. It is estimated that approximately 13% of arable land and 40% of lakes and rivers worldwide are contaminated with heavy metals. Although cadmium is a widespread environmental pollutant with well-established toxicity, the full extent of its effects on human health remains unclear [10]. Because of their carcinogenic properties, cadmium and cadmium compounds have been classified by the International Agency for Research on Cancer (IARC) as Group 1 human carcinogens [11].

According to the WHO guidelines for cadmium, the provisional tolerable monthly intake was established at 25 µg/kg body weight on the basis of a meta-analysis of epidemiological studies examining the association between urinary cadmium and beta-2-microglobulin, a marker of renal tubular effects. The guideline value for cadmium in drinking water is 3 µg/L, whereas the air-quality guideline is 5 ng/m^3^ as an annual mean [12]. One of the characteristics that makes Cd particularly harmful is its long cumulative half-life. Following oral intake, it accumulates primarily in the kidneys and liver; following inhalation, it is transported to the lungs. Its mechanisms of toxicity are associated with reactive oxygen species (ROS) and inflammation [13].

Cd causes toxic effects in several organs, particularly the kidneys, bones, cardiovascular system, and nervous system. The manifestations of Cd poisoning may vary according to the timing of exposure, dietary patterns, and the age and health status of exposed individuals. Studies have evaluated associations between Cd exposure and a range of health outcomes, including, but not limited to, chronic kidney disease, cardiovascular disease, cancer, diabetes, and impaired cognitive and behavioral function. Accordingly, investigating the relationship between cadmium exposure and health outcomes is of major public health importance [14]. Daily Cd intake attributable to cigarette exposure has been estimated at 0.0359 µg/kg body weight/day. The proportions of the burdens of cardiovascular disease (CVD), stroke, and coronary heart disease (CHD) attributable to dietary Cd have been estimated at 6.08%, 3.34%, and 2.74%, respectively [15]. Cd may contribute to carcinogenesis by affecting several signaling pathways, including ERK/JNK/p38 MAPK, PI3K/AKT/mTOR, NF-κB, and Wnt. It can also induce DNA damage, disrupt DNA-repair mechanisms, and contribute to the development of various diseases through epigenetic mechanisms such as aberrant DNA methylation, long noncoding RNAs (lncRNAs), and microRNAs (miRNAs) [2].

### 1.2. Significance of miRNAs

MicroRNAs (miRNAs) are well-characterized noncoding RNAs that regulate gene expression at the post-transcriptional level [16]. As one of the principal epigenetic regulatory mechanisms, miRNAs are regarded as alternative biomarkers for various human diseases [17]. Evolutionarily conserved small noncoding RNAs (sncRNAs) have been investigated for their roles in regulating protein translation and contributing to disease pathophysiology [18]. MiRNAs are small, single-stranded, endogenous noncoding RNA molecules that regulate gene expression post-transcriptionally by binding to complementary sequences within the 3′ untranslated regions (3′ UTRs) of target mRNAs. This interaction can result in translational repression or mRNA degradation, and altered miRNA expression may contribute to the development of numerous human diseases by participating in pathophysiological processes such as inflammation, apoptosis, and autophagy [19,20]. MiRNAs have also been reported to function as critical epigenetic regulators of cellular growth and development, including cell differentiation, proliferation, migration, invasion, cell-cycle progression, and stress responses [21]. MiRNAs and long noncoding RNAs (lncRNAs) can be positively or negatively regulated by epigenetic regulators such as EZH2. Heavy metals are known not only to alter protein-expression profiles but also to affect miRNAs through the regulation of epigenetic markers [22]. Various environmental hazards have been shown to alter miRNA-expression profiles. By inducing epigenetic changes that affect miRNA biogenesis and function, these exposures may influence cellular stress responses and diverse disease processes [23,24].

### 1.3. Relationship Between Cadmium and miRNAs

Hundreds of lncRNAs and miRNAs have been confirmed to be associated with Cd toxicity [25]. The mechanisms underlying Cd-mediated toxicity have been extensively investigated over the past several decades. Interactions between Cd and key cellular sites lead to the generation of reactive oxygen species (ROS), resulting in DNA damage, lipid peroxidation, antioxidant depletion, and alterations in calcium (Ca) homeostasis [26]. Although Cd-associated changes in DNA methylation have been described in detail, the role of other epigenetic regulatory mechanisms, such as miRNAs, in cadmium toxicity has not yet been fully elucidated [27].

Studies indicate that cadmium exposure can alter miRNA-expression profiles. In individuals occupationally exposed to cadmium, miR-146a was reported to be downregulated and miR-222 upregulated, with these changes being associated with cytokine levels related to the immune response [28]. Environmental exposures have been shown to modify miRNA-expression profiles, and exposure-related miRNA alterations may affect cellular processes such as inflammation, oxidative stress, apoptosis, and immune signaling [24]. MiRNAs are regulatory molecules that are sensitive to environmental factors. Heavy-metal toxicity has been reported to involve miRNA-mediated mechanisms. Accordingly, dysregulated miRNA expression is considered a potential biomarker of chronic metal exposure [29]. Cd exposure has been reported to alter the hepatic miRNA-expression profile, with the target genes of differentially expressed miRNAs enriched in biological processes related to inflammation and apoptosis, particularly the MAPK and TNF signaling pathways [30]. Consistent with these observations, cadmium has been shown to induce cellular apoptosis by activating the mitochondrial apoptotic pathway through ROS/JNK signaling [31]. In addition, cadmium exposure has been shown to increase the expression of proinflammatory cytokines, including TNF-α, IL-1, and IL-6, and to enhance hepatocyte apoptosis [32].

Several narrative reviews have already synthesized aspects of the cadmium–miRNA literature. Koushki et al. (2024) [29] provided a comprehensive qualitative review specifically of cadmium-induced miRNA alterations; Wallace et al. (2020) [33] reviewed miRNA expression changes across toxic metals more broadly, of which cadmium was one example; and Chaiwangyen et al. (2025) [24] reviewed miRNA responses to environmental hazards in general, a scope considerably broader than cadmium alone. None of these three reviews applied a systematic, multi-database bibliometric search strategy, quantified keyword co-occurrence or citation structure, or performed formal statistical testing (exact confidence intervals, sensitivity analyses, or network null models) of cross-study directional consistency. The present study does not claim topical novelty in identifying that cadmium alters miRNA expression, a relationship already well established by these reviews; its contribution is instead methodological: a reproducible bibliometric quantification of the field’s structure and growth (Section 2.1, Section 2.2, Section 2.3 and Section 2.4), combined with an explicitly separated, statistically tested direction-consistency analysis (Section 2.5) and a shared-target network analysis with a null-model test of hub significance (Section 2.6 and Section 2.7) that, to our knowledge, have not been previously applied to this literature.

In recent years, an increasing number of studies have investigated the role of miRNA-mediated mechanisms in heavy-metal toxicity [33]. Nevertheless, although the regulatory roles of miRNAs in various cellular processes have been characterized, their functions in specific pathological mechanisms and the molecular mechanisms underlying certain miRNA-mediated apoptotic processes remain under investigation [34]. Therefore, although qualitative reviews of this relationship exist, a quantitative, reproducible bibliometric assessment of research trends, prominent research topics, and the biological processes associated with the most frequently reported miRNAs remains limited. This study aimed to examine publications on cadmium and miRNAs using bibliometric methods, identify prominent miRNAs, and evaluate their shared targets and potential roles in oxidative stress, immune responses, and apoptosis through bioinformatic and network analyses.

## 2. Results

### 2.1. Keyword Co-Occurrence and Thematic Structure

**Figure 1:**
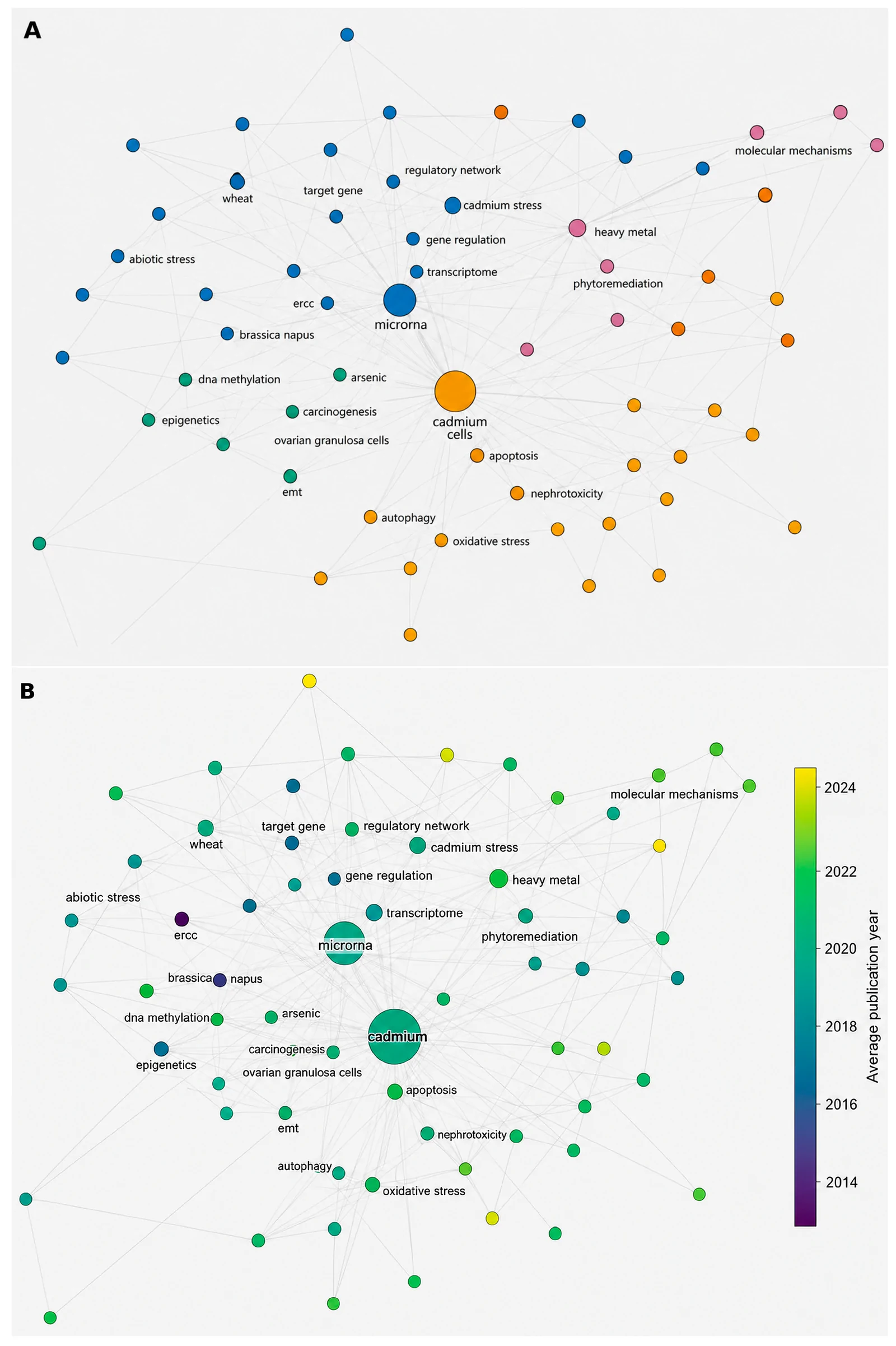

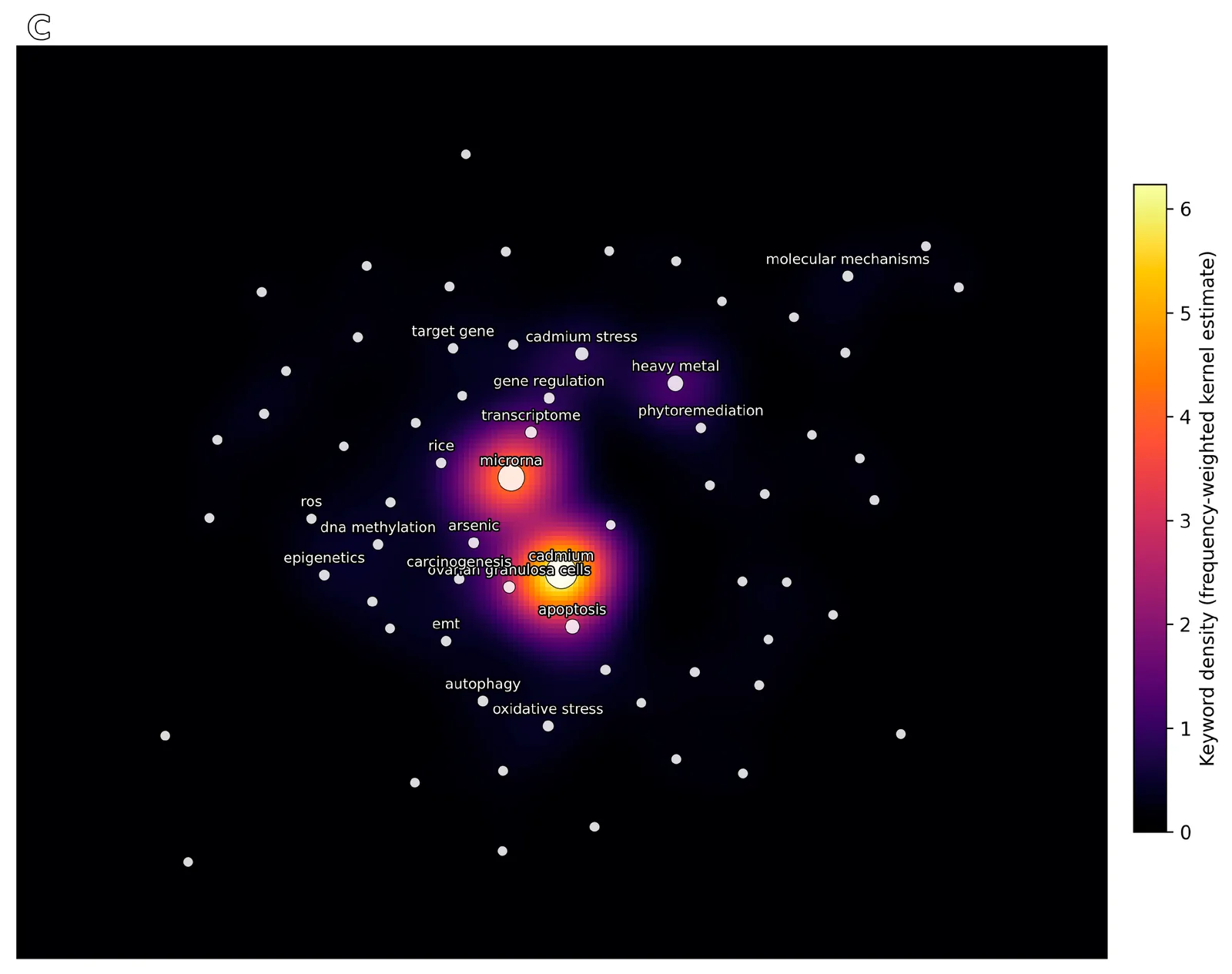
Author-keyword co-occurrence analysis of the final multi-database bibliometric corpus (*n* = 286) at a minimum occurrence threshold of 3 (67 keywords, 261 links, total link strength = 610). (**A**) Network visualization, where node size indicates keyword frequency, edge width indicates co-occurrence strength, and node color denotes five thematic clusters: Cluster 1, miRNA/plant stress response; Cluster 2, cadmium/apoptosis/oxidative stress; Cluster 3, epigenetics/carcinogenesis; Cluster 4, heavy metal/molecular mechanisms; and Cluster 5, signal transduction/other. Colors correspond to Clusters 1–5 as blue, gold, green, magenta, and orange, respectively. (**B**) Temporal overlay visualization of the same keyword network, where node color indicates the mean publication year of each keyword. The same node layout is retained in panels (**A**,**B**) to facilitate direct comparison between thematic clustering and temporal distribution. (**C**) Density visualization of the same keyword network, where brighter regions indicate higher local keyword density weighted by occurrence frequency. Explanatory cluster descriptions are provided in the figure legend and Section 2.1 rather than repeated within the graphics. A minimum occurrence threshold of 5 (25 keywords, 81 links, total link strength = 313) was evaluated as a sensitivity analysis and is reported in Section 2.1.

Using the final multi-database bibliometric corpus (*n* = 286), author-keyword co-occurrence analysis was performed after basic thesaurus-based harmonization (merging trivial spelling/plural variants; full mapping supplied in the accompanying submission reproducibility package). In the primary analysis, a minimum occurrence threshold of ≥3 retained 67 keywords, all belonging to a single connected network comprising 261 links and a total link strength of 610 (Figure 1A). A stricter minimum occurrence threshold of ≥5 was used as a prespecified sensitivity analysis; 25 keywords remained connected through 81 links with a total link strength of 313.

At the ≥3 threshold, “cadmium” was the most frequently occurring author keyword (152 occurrences; total link strength = 235), followed by “microRNA” (106; 212), “heavy metal” (29; 43), “apoptosis” (21; 45), and “cadmium stress” (17; 35). The same five terms remained the most frequent keywords in the ≥5 sensitivity analysis, indicating that the principal thematic core was robust to removal of lower-frequency terms.

The network showed a concentrated thematic structure organized into five keyword clusters (VOSviewer version 1.6.18 clustering; Figure 1A). Cadmium and microRNA regulation formed the dominant core, while heavy-metal toxicity, apoptosis, and cadmium-stress responses represented the most recurrent secondary themes. Epigenetics and DNA methylation were represented within a distinct carcinogenesis-related cluster, and epithelial–mesenchymal transition (EMT) was retained at the ≥3 threshold within this same cluster.

The temporal overlay visualization suggested a shift toward more recent mechanistic and regulatory themes (Figure 1B). The terms “molecular mechanisms” (mean publication year 2023.1) and “lncRNA” (2024.0) were positioned toward more recent average publication years, whereas “epigenetics” (2017.4) occupied an older region of the temporal map, consistent with a shift in research emphasis over the study period. The density visualization showed the highest concentration around cadmium and microRNA, with secondary density extending toward heavy metal and apoptosis (Figure 1C).

### 2.2. Co-Authorship Network Analysis

**Figure 2:**
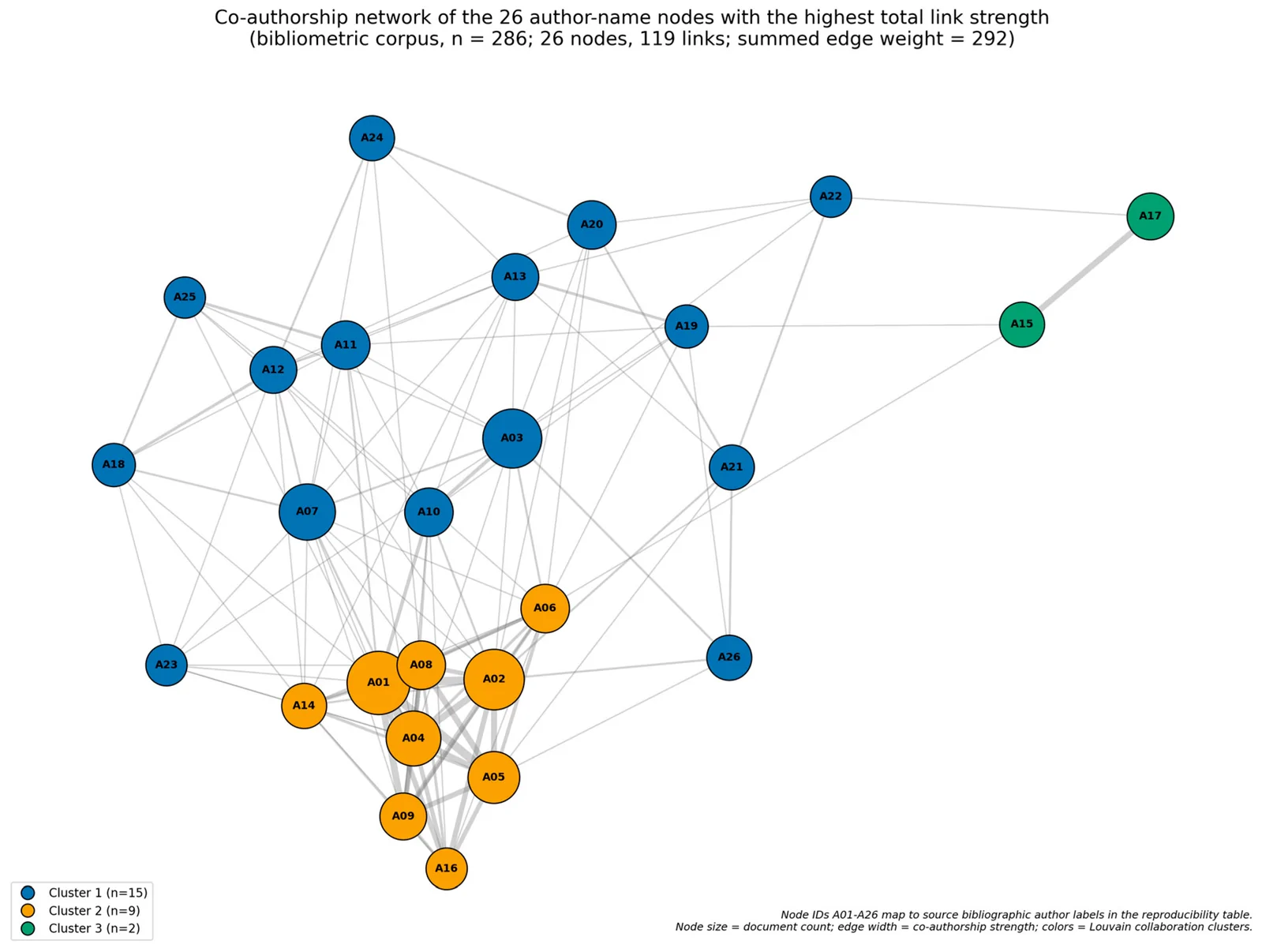
Co-authorship network of the 26 author-name nodes with the highest full-graph total link strength in the final bibliometric corpus (*n* = 286; 283 author-name labels met the ≥2-document retention criterion out of 1234 unique exported labels). The induced 26-node network comprises 119 links with a summed edge weight of 292 and resolves into three Louvain collaboration clusters (15, 9, and 2 nodes; resolution = 1.00; seed = 42). Because merged database exports use surname–initial labels that can conflate homonymous authors, analytical node IDs A01–A26 are used rather than unsupported person-level assignments. For clarity, specific source records underlying abbreviated labels include Lili Wang and Yangyi Zhang in Zhang et al. (2021) [35] and Jiabin Chen in Chen et al. (2021) [36]; these examples clarify the source labels but are not assumed to establish a one-to-one identity for the aggregated nodes. The full source-label mapping is provided in the reproducibility table. Node size corresponds to document count and edge width to co-authorship strength.

Co-authorship analysis was performed to characterize scientific collaboration within the final multi-database bibliometric corpus. Across the 286 included publications, 1234 unique exported author-name labels were identified. To focus the network on recurring contributors, labels occurring in at least two documents were retained; 283 met this criterion. The 26 author-name labels with the greatest full-graph total link strength were selected for visualization. Because the source exports use abbreviated bibliographic labels rather than validated person identifiers, the displayed nodes were assigned analytical IDs A01–A26 in Figure 2; the source-label mapping is supplied in the reproducibility table. Full-name source examples and their references are provided below to clarify the abbreviated labels without implying a one-to-one identity for aggregated nodes.

The 26 selected author-name nodes formed a single connected co-authorship network comprising 119 links with a summed edge weight of 292 (Figure 2). The network resolved into three collaboration clusters containing 15, 9, and two nodes. Within the visualized subgraph, the largest weighted degrees were observed for A01 (TLS = 70), A02 (TLS = 57), A04 (TLS = 53), A05 (TLS = 51), A08 (TLS = 42), A09 (TLS = 40), and A16 (TLS = 32). These node IDs are analytical identifiers only and are not asserted to represent uniquely disambiguated individuals.

Because the merged bibliographic exports contain abbreviated author-name strings and common surname–initial combinations may map to more than one individual, the co-authorship network is interpreted at the author-name-node and collaboration-cluster level. Source verification identified Lili Wang and Yangyi Zhang in Zhang et al. (2021) [35] and Jiabin Chen in Chen et al. (2021) [36] as full-name instances associated with abbreviated labels appearing in the underlying corpus. However, these specific source matches do not justify treating an aggregated surname–initial node as a uniquely disambiguated person. Figure 2 therefore emphasizes collaboration structure and recurrent co-authorship patterns rather than person-level brokerage.

### 2.3. Recurrently Reported miRNAs and Direction of Expression Changes

Among the studies included in the final human-relevant biological evidence corpus, miR-155 was the most frequently recurrent miRNA and was reported in four studies. The let-7 family, miR-146b, miR-222, and the miR-30 family were each reported in three studies. None of these recurrent miRNAs showed a uniformly replicated cross-study direction. Instead, the let-7 family and miR-30 family showed a modest predominance of decreased expression, whereas miR-146b and miR-222 showed a modest predominance of increased expression. miR-155 displayed the greatest directional dispersion, with two studies reporting increased expression and two reporting decreased expression. As a sensitivity check on the ≥3-study inclusion threshold used to define recurrence, applying a stricter ≥ 4-study threshold would retain only miR-155 (*n* = 4); the let-7 family, miR-146b, miR-222, and the miR-30 family (each *n* = 3) would no longer qualify as recurrent under this stricter criterion, underscoring that the recurrence status of all miRNAs other than miR-155 depends on where this threshold is set.

Taken together, the recurrent miRNAs were linked mainly to inflammatory and stress-response processes, epithelial or renal injury contexts, and broader cadmium-associated adaptive responses. However, the mixed direction of expression changes across studies indicates substantial biological heterogeneity related to model system, specimen type, exposure dose and duration, and study design (Table 1).

### 2.4. Publication Trends and Highly Cited Studies

**Figure 3:**
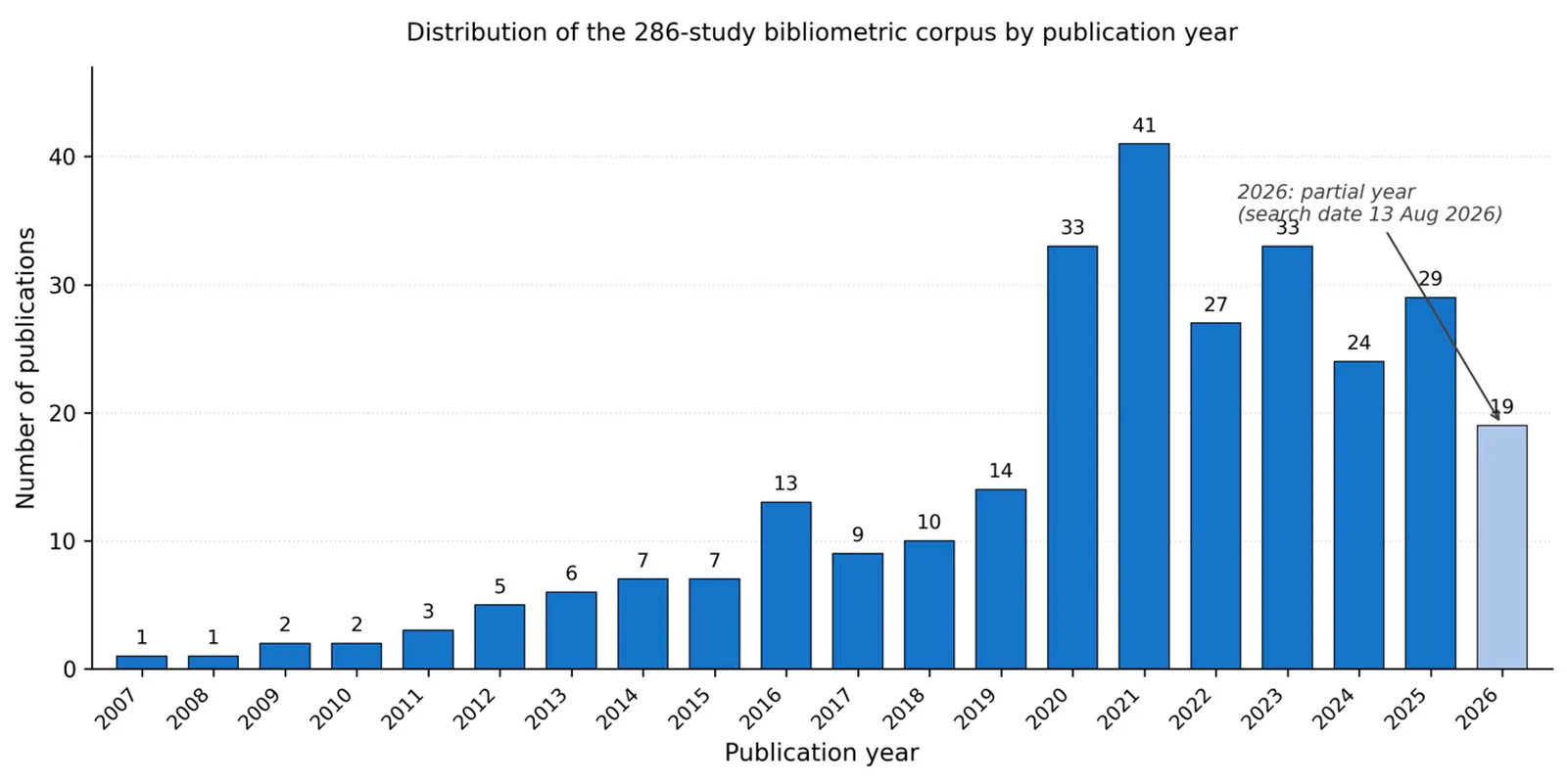
Distribution of the 286-study bibliometric corpus by publication year (2007–2026). The 2026 bar (light shading) reflects a partial year only, as the literature search was conducted on 13 August 2026.

The annual distribution of the 286-study bibliometric corpus demonstrated a marked increase in research activity over time (Figure 3). The first eligible study was published in 2007, and publication output remained low (fewer than 15 studies per year) through 2019. From 2020 onward, output rose sharply: 206 of the 286 studies (72%) were published in 2020 or later. The highest number of publications was recorded in 2021 (41 studies), followed by 2020 and 2023 (33 studies each) and 2025 (29 studies). The 2026 count (19 studies) reflects a partial year only, as the literature search was conducted on 13 August 2026, and should not be interpreted as a decline in research activity. This temporal pattern indicates rapidly growing scientific interest in the epigenetic effects of cadmium, particularly in miRNA-mediated regulatory mechanisms, especially over the past six years.

Among the most highly cited publications, the study by Zhang et al. (2021) [35], which investigated the lncRNA OIP5-AS1/miR-128-3p/SLC7A11 regulatory axis in the context of long-term cadmium exposure, ranked first with 167 citations and a field-weighted citation impact of 9.15 (Table 2). This was followed by the study by Hassan et al. (2012) [37], which examined the roles of miR-101 and miR-144 in regulating CFTR chloride-channel expression and received 140 citations, and the review by Wang et al. (2012) [38], which evaluated the epigenetic effects of cadmium and received 138 citations.

Other prominent miRNAs represented among the five most highly cited studies included miR-365 and members of the let-7 family. Overall, the most influential publications were predominantly focused on epigenetic regulation, oxidative-stress responses, ferroptosis-related mechanisms, and miRNA-mediated molecular pathways associated with cadmium exposure.

### 2.5. Cross-Study Directionality and Heterogeneity

**Figure 4:**
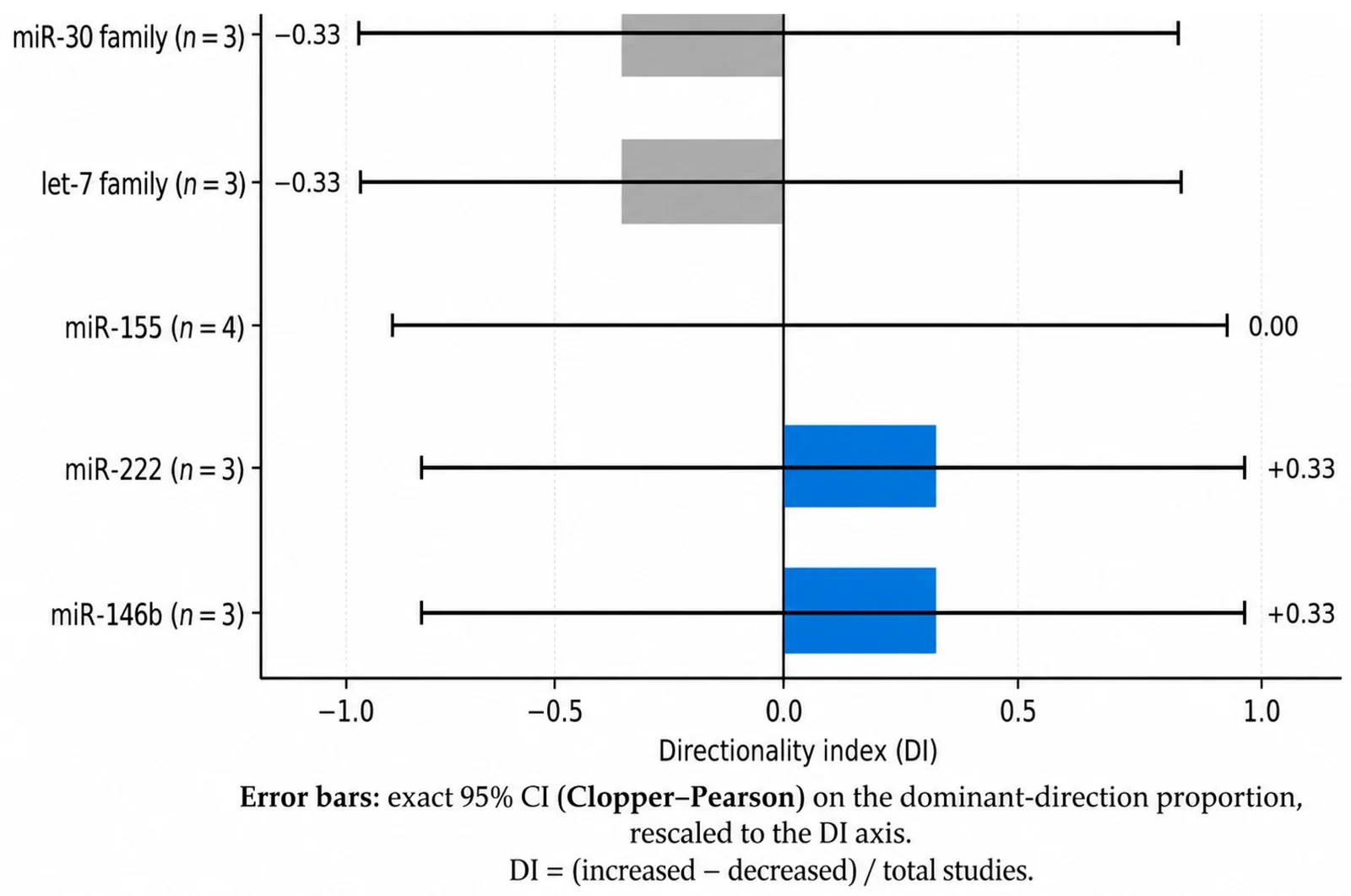
Cross-study directionality index of recurrent cadmium-associated miRNAs. Positive values indicate a predominance of increased expression, whereas negative values indicate a predominance of decreased expression. Gray bars indicate negative directionality indices, whereas blue bars indicate positive directionality indices. Error bars show the exact (Clopper–Pearson) 95% confidence interval for the dominant-direction proportion, rescaled to the directionality-index axis; *n* indicates the number of studies contributing a directional vote for each miRNA. Intervals crossing zero indicate that the observed dominant direction is not statistically distinguishable from a 50:50 split at this sample size.

Direction-consistency analysis based on the final biological evidence corpus showed that no recurrent miRNA demonstrated a robust, uniformly replicated direction across four or more studies. miR-155 was the most frequently reported recurrent miRNA, but it showed a balanced pattern, with two studies reporting increased expression and two reporting decreased expression (DI = 0.00; dominant-direction proportion = 0.50; H = 0.631). The let-7 family and the miR-30 family each showed a modest downward tendency (one increased and two decreased; DI = −0.33), whereas miR-146b and miR-222 each showed a modest upward tendency (two increased and one decreased; DI = +0.33). Overall, these findings support directional heterogeneity rather than stable cross-study concordance in the final biological evidence corpus (Table 3 and Figure 4).

To formally test whether these dominant-direction proportions exceed what would be expected by chance, we computed exact (Clopper–Pearson) 95% confidence intervals and two-sided exact binomial *p*-values against a 50:50 null for each recurrent miRNA (*n* = studies contributing a directional vote; Table 3). None of the five recurrent miRNAs showed a statistically significant departure from a 50:50 split (all *p* = 1.00; 95% CIs spanning approximately 7–93% for miR-155 and 9–99% for the remaining four miRNAs), confirming that the observed dominant-direction proportions of 0.50–0.67 are fully compatible with chance at these small sample sizes. We additionally performed a sensitivity analysis collapsing studies from the same research group and model system into a single evidence unit (Research_Group_Model_ID); for all five recurrent miRNAs, every contributing study belonged to a distinct research group and model system, so the collapsed analysis returned identical counts to the study-level analysis (0 votes removed), indicating that the reported patterns are not an artifact of repeated observations from the same laboratory or cell line. Raising the inclusion threshold from ≥3 to ≥4 studies retained only miR-155, underscoring that the ≥3 threshold used here is a deliberately low bar intended to surface candidate patterns rather than establish reproducibility.

A plausible alternative explanation for this directional heterogeneity is technical rather than purely biological. The ten studies contributing votes to the five recurrent miRNAs differed substantially in exposure matrix, model system, quantification platform, dose or exposure metric, and normalization strategy (Table 4). Most used human-derived in vitro models, whereas Mitra et al. [28] and Zheng et al. [41] also contributed human exposure-cohort data. Exposure conditions ranged from acute 24–72 h micromolar CdCl2 treatments to chronic cadmium transformation models and occupational or environmental exposure. Primary-source verification confirmed an explicit normalization strategy for six of the ten studies (Fabbri et al., 2012 [40]; Ngalame et al., 2016 [42]; Lemaire et al., 2020 [43]; Brooks et al., 2016 [44]; Mortoglou et al., 2022 [45]; and Tanwar et al., 2019 [46]), while normalization remained unconfirmed from accessible primary text for the remaining studies. Because technical reporting remains incomplete and heterogeneous, we did not formally model expression direction as a function of matrix, platform, or dose. The same verification was applied to the four-criterion methodological-completeness assessment across the final 32-study biological evidence corpus (dose/exposure, platform, normalizer, and sample size/replicate structure; Supplementary Table S1): five of 32 studies (15.6%) reported none of the four criteria explicitly, whereas nine of 32 (28.1%) reported all four. These findings reinforce the need for cautious interpretation of any apparent dominant direction and for standardized exposure and miRNA-reporting procedures across future studies.

### 2.6. Exploratory Candidate-miRNA–Target Network Architecture and Shared Target Hubs

The exploratory mechanism-oriented network comprised 10 candidate miRNA nodes, 51 unique target-gene nodes, and 66 miRNA–target edges distributed across four connected components. This network is analytically separate from the five-miRNA recurrence/directionality analysis in Section 2.3 and Section 2.5: it includes candidate miRNAs for which sufficiently supported arm-specific, experimentally validated human target sets could be curated from the human-relevant mechanistic literature. Accordingly, inclusion in this network should not be interpreted as evidence that a miRNA met the final cross-study recurrence threshold. hsa-miR-155-5p had the largest target degree (10), followed by hsa-miR-21-5p (9). hsa-miR-222-3p and the miR-30 family each had seven target connections; hsa-miR-126-3p, hsa-miR-221-3p, and hsa-miR-26a-5p each had six; and hsa-miR-146a-5p, hsa-miR-146b-5p, and hsa-miR-26b-5p each had five. These degrees reflect the deliberately restricted mechanism-oriented target set and should not be interpreted as the total number of biological targets for each miRNA.

**Figure 5:**
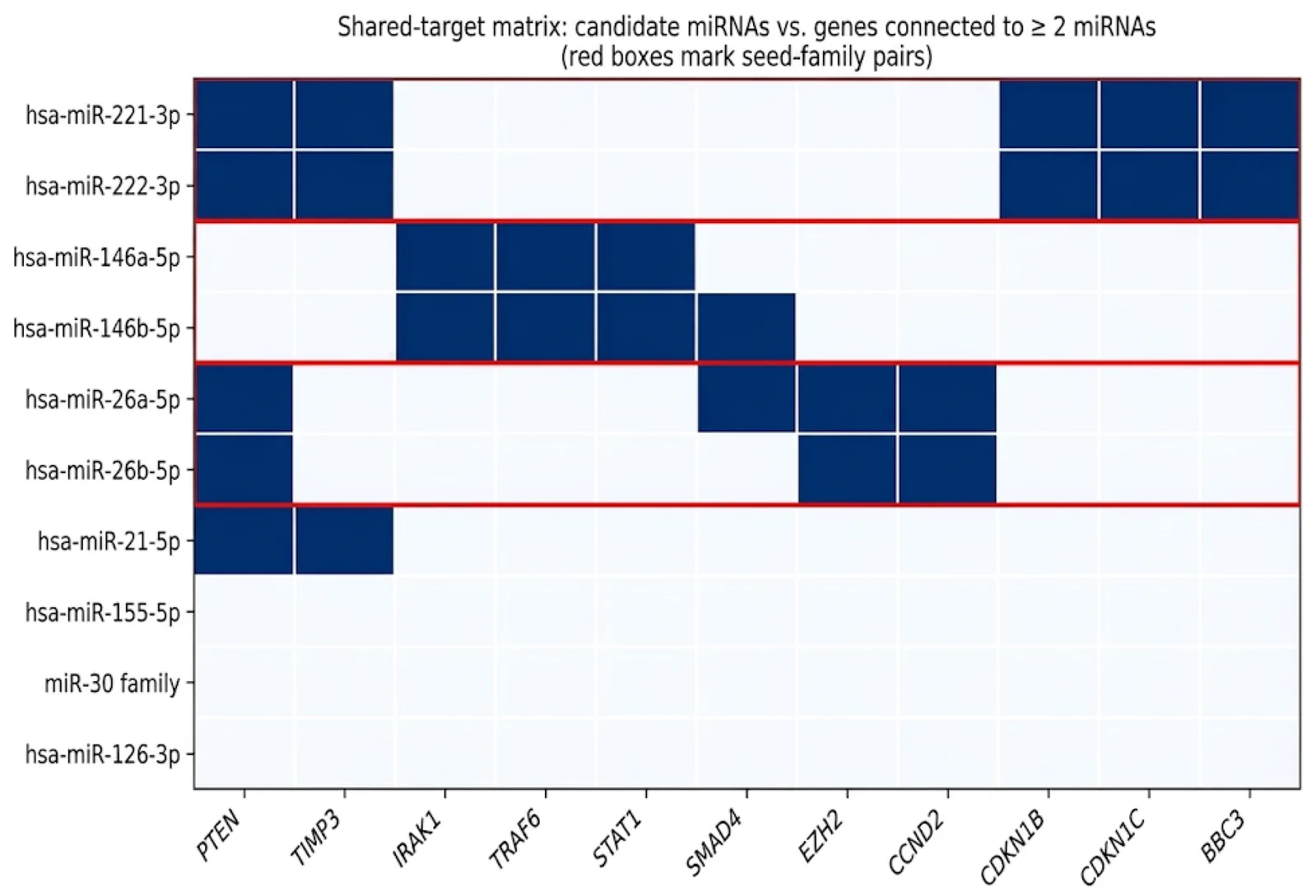
Shared-target binary matrix restricted to genes connected to at least two candidate miRNAs in the exploratory mechanism-oriented network. Filled cells indicate a curated miRNA–target connection; rows are ordered so that the three seed–family pairs (hsa-miR-221-3p/hsa-miR-222-3p; hsa-miR-146a-5p/hsa-miR-146b-5p; hsa-miR-26a-5p/hsa-miR-26b-5p, outlined in red) are directly adjacent, illustrating that most shared-target connections are concentrated within these paralog pairs rather than distributed broadly across unrelated miRNAs.

*PTEN* was the most prominent shared target and was connected to five miRNAs: hsa-miR-21-5p, hsa-miR-26a-5p, hsa-miR-26b-5p, hsa-miR-221-3p, and hsa-miR-222-3p. TIMP3 was connected to three miRNAs, whereas *BBC3*, *CCND2*, *CDKN1B*, *CDKN1C*, *EZH2*, *IRAK1*, *SMAD4*, *STAT1*, and *TRAF6* were each connected to two. Pairwise overlap analysis identified the strongest co-targeting relationship between hsa-miR-221-3p and hsa-miR-222-3p, which shared five targets (*BBC3*, *CDKN1B*, *CDKN1C*, *PTEN*, and *TIMP3*; Jaccard coefficient = 0.625). Hsa-miR-146a-5p and hsa-miR-146b-5p shared IRAK1, STAT1, and *TRAF6* (Jaccard coefficient = 0.429), whereas hsa-miR-26a-5p and hsa-miR-26b-5p shared *CCND2*, *EZH2*, and *PTEN* (Jaccard coefficient = 0.375). The shared-target subnetwork is shown in Figure 5, and the complete curated target sets used in the analysis are listed in Table 5.

Because hsa-miR-221-3p/hsa-miR-222-3p, hsa-miR-146a-5p/hsa-miR-146b-5p, and hsa-miR-26a-5p/hsa-miR-26b-5p are seed–family paralogs, elevated target overlap within each pair is an expected consequence of shared seed-sequence biology and does not by itself constitute independent evidence of cadmium-specific convergence. To assess this directly, we distinguished within-family from cross-family miRNA pairs in the curated network. The 42 possible cross-family (non-seed-sharing) pairs had a mean Jaccard coefficient of only 0.022, and 33 of 42 (79%) shared no target at all, compared with a mean of 0.476 for the three within-family pairs. A random-reassignment null model (20,000 iterations; Section 4.7), in which each miRNA’s observed number of curated targets was held fixed but the specific target genes were redrawn uniformly at random from the same 51-gene curated pool, confirmed that all three within-family Jaccard values exceed chance expectation even after conditioning on target-set size and the shared gene pool (*p* = 0.0001–0.0084). Under the same null model, *PTEN*’s observed degree of five was higher than expected for that specific gene (*p* = 0.004), although reaching a maximum degree of five by some gene in the network was not itself unusual (*p* = 0.23), indicating that a hub of this size is not surprising per se, but *PTEN*’s particular recurrence across independent miRNA–target sets is unlikely to be due to chance alone. Together, these analyses indicate that the network’s densest connections are concentrated within seed–family pairs and around *PTEN*, rather than reflecting broad co-targeting across unrelated miRNA families.

### 2.7. Descriptive Functional Annotation of the Curated Target Network

Knowledge-guided functional annotation grouped the curated target genes into eight process-level modules (Table 6). Because the underlying target set was itself assembled using a prespecified, cadmium-relevance-oriented curation filter (Section 4.7) rather than an unbiased or exhaustive target universe, this modular structure should be read as a descriptive annotation of the curated evidence set rather than as independent evidence that cadmium-responsive miRNAs converge on these processes genome-wide. The innate immune/TLR-NF-κB module contained 13 genes and was connected to four miRNAs, particularly the miR-146a/b and miR-155 axes. TGF-beta/EMT, extracellular-matrix remodeling, and fibrosis formed a 10-gene module connected to six miRNAs. PI3K-AKT and growth-factor signaling comprised eight genes and six miRNAs, with *PTEN* recurring as the most frequently shared target (Section 2.6). Apoptosis and cell-cycle regulation involved eight genes and seven miRNAs. Oxidative-stress regulation involved *BACH1*, *FOXO3*, *PTEN*, and *PTGS2* and was connected to six miRNAs. Endothelial/angiogenic regulation involved seven genes and three miRNAs, with miR-126-3p contributing the largest proportion of targets. Additional modules included epigenetic regulation and DNA repair (*DNMT3A*, *EZH2*, and *MSH2*) and autophagy (*ATG5* and *BECN1*). The module-level results are summarized in Table 6.

Taken together, this descriptive annotation indicates that the curated target genes are distributed across several process-level modules rather than a single linear pathway, spanning inflammatory feedback, redox homeostasis, survival signaling, cell-cycle control, autophagy, endothelial function, and tissue-remodeling programs. This breadth is, at least in part, a direct consequence of how the target set was assembled: edges were retained only when a target gene was judged relevant to at least one prespecified cadmium-associated process (Section 4.7), so the network’s apparent coverage of multiple processes partly reflects the curation criteria themselves rather than an independent, unbiased survey of miRNA targets. Because the network was constructed from a conservative, investigator-curated evidence set rather than an exhaustive or unbiased transcriptome-wide target list, these module-level results should be interpreted as a structured summary of the curated literature and as prioritized, hypothesis-generating candidates for experimental validation, not as statistical evidence of pathway enrichment or convergence.

## 3. Discussion

This study combined multi-database bibliometric mapping, structured content review, cross-study directionality analysis, and an exploratory miRNA–target network to characterize the developing literature on cadmium exposure and miRNA regulation. Three principal findings emerged. First, the use of PubMed/MEDLINE, Scopus, and Web of Science materially broadened coverage and yielded a final bibliometric corpus of 286 topic-relevant publications. Second, the keyword analysis produced a more concentrated thematic structure at occurrence thresholds of ≥3 and ≥5, with cadmium and microRNA forming the dominant core and heavy-metal toxicity, apoptosis, and cadmium-stress responses remaining stable secondary themes. Third, the final biological evidence corpus did not support a uniformly reproducible direction for any recurrent miRNA; instead, the available studies showed substantial context-dependent heterogeneity, with miR-155 displaying a balanced up/down pattern and the remaining recurrent signals showing only modest directional tendencies.

The multi-database bibliometric corpus indicates that the cadmium–miRNA field has grown from early expression-profiling studies toward more mechanistic work involving noncoding-RNA networks and stress-response pathways. In the author-keyword analysis at the ≥3 occurrence threshold, epigenetics and circRNA remained represented but were peripheral relative to the cadmium–microRNA core, whereas circRNA and lncRNA appeared among the more recent terms in the temporal overlay. This pattern suggests an evolution toward increasingly network-oriented mechanistic models without implying that any single emerging topic defines the field. The use of stricter occurrence thresholds also reduced the influence of low-frequency peripheral terms and produced a more stable thematic core [24,29,33,35].

A central finding of the biological synthesis is that recurrence should not be equated with directional reproducibility. In the final biological evidence corpus, miR-155 was reported in four studies but showed an equal number of increases and decreases (2/2). The let-7 family and miR-30 family each showed one increase and two decreases, whereas miR-146b and miR-222 each showed two increases and one decrease. With only three to four contributing studies per recurrent miRNA, these patterns remain imprecise and are compatible with substantial uncertainty. Accordingly, the observed directions are described as limited, study-level tendencies rather than as ‘uniform’, ‘consistent’, or ‘reproducible’ responses.

The miR-155 findings illustrate the extent to which cadmium-associated miRNA responses may depend on experimental and analytical context. Its balanced up/down pattern argues against treating miR-155 as a unidirectional exposure marker. Differences among studies may reflect biological factors such as cell type, tissue, dose, duration, chronicity, and disease context, but methodological factors are also important, including RNA extraction, assay platform, normalization strategy, sample size, and the definition of statistical significance. miR-146b and miR-222 showed modest upward tendencies in the recurrent set, but these signals are based on only three studies each and should therefore be interpreted cautiously rather than as evidence of stable induction. Representative human and human-derived studies illustrate this context dependence [28,45,48].

The evidence hierarchy also separates recurrent expression evidence from mechanistic relevance. miR-222 remains recurrent in the final directionality dataset, whereas miR-221 does not currently meet the same recurrence threshold under the stricter study-level rules. Consequently, the available evidence does not support a coherent miR-221/222 directional signal as a cross-study finding. Mechanistic interactions involving miR-221/222 and targets such as *PTEN*, *TIMP3*, *CDKN1B*, *CDKN1C*, and *BBC3* [49] may still be biologically informative, but shared targets within seed-related miRNA families should not be interpreted as independent evidence of coordinated cadmium-specific regulation. This caution is reinforced by the wider miRNA–target literature: *PTEN* is among the most extensively validated microRNA targets reported across human disease research in general, with a systematic review documenting *PTEN* as a recurrent target of the miR-221/222 family alone across more than a dozen non-cadmium cancer and disease contexts [49]. *PTEN*’s hub status in the present curated network is therefore more parsimoniously explained by its general connectivity as a frequently validated miRNA target than by cadmium-specific regulatory convergence, consistent with the degree-preserving null-model result reported above (Section 2.6). These network observations are therefore treated as hypothesis-generating annotations pending background-controlled evaluation.

Similarly, miR-126 no longer meets the recurrence threshold in the final expression dataset and should not be described as having a reproducible downward response to cadmium. Its established links to endothelial and angiogenic regulation through targets such as *PIK3R2*, *SPRED1*, *VCAM1*, *IRS1*, *CRK*, and *EGFL7* [50] remain mechanistically relevant, particularly in the context of cadmium-associated vascular injury, but this biological plausibility is distinct from cross-study directional evidence. Future validation should therefore evaluate miR-126 in well-characterized human cohorts with simultaneous measures of cadmium exposure, vascular biomarkers, and standardized miRNA quantification. The cadmium-responsive miR-126 signal in pancreatic ductal adenocarcinoma cells provides mechanistic context but does not by itself establish cross-study reproducibility [45].

Among the recurrent signals, the miR-30 family showed a modest downward tendency, while miR-155, miR-146b, miR-222, and the let-7 family showed differing patterns across studies. Other miRNAs, including miR-21 and the miR-26 family, remain relevant in individual mechanistic reports but do not satisfy the final recurrence criterion. This distinction is important because biological plausibility, target-network connectivity, and recurrence across independent studies are separate dimensions of evidence. The interpretation therefore prioritizes transparent study-level reporting over assigning broad pathway meaning to miRNAs that are not recurrent under the final study-level extraction rules.

Oxidative stress remains an important biological context across the cadmium–miRNA literature, but the present analysis does not treat it as proof of a single convergent miRNA mechanism. Highly cited mechanistic studies have described noncoding-RNA axes involving OIP5-AS1/miR-128-3p/*SLC7A11* and *MT1DP*/miR-365/NRF2, while newer bibliometric terms point toward ferroptosis and ceRNA-related work. These observations support continued investigation of redox-sensitive miRNA mechanisms, but pathway-level conclusions should be based on unbiased target collection and formal enrichment rather than on a preselected mechanism-oriented gene set [25,30,35].

From a biomarker perspective, the present findings argue strongly against relying on a single miRNA across heterogeneous cadmium-exposure settings. None of the recurrent miRNAs shows sufficiently robust directional evidence to support stand-alone biomarker claims. A future multi-miRNA panel may be more informative, but candidate selection should incorporate recurrence, effect size, biological matrix, exposure metric, assay platform, normalization strategy, and external validation rather than direction counts alone. Population-based translation will additionally require careful control of smoking, diet, renal function, occupational co-exposures, age, sex, inflammatory status, and pre-analytical sample handling. This cautious interpretation is consistent with recent cadmium–miRNA and environmental-miRNA reviews [24,29,33].

This study has several methodological strengths. The literature search spans three complementary databases, cross-database duplicates are explicitly resolved, and the bibliometric corpus is separated from the primary biological evidence corpus. Keyword mapping is evaluated at both ≥3 and ≥5 occurrence thresholds, and the biological synthesis uses a study-level extraction framework that distinguishes direct cadmium-related expression evidence from association-only and mechanistic evidence. The directionality analysis is therefore intended as a transparent descriptive synthesis with explicit recognition of small sample sizes and study dependence, rather than as a substitute for effect-size meta-analysis.

Several limitations remain. First, although the search included PubMed/MEDLINE, Scopus, and Web of Science Core Collection, it was restricted to English-language journal literature and may still miss eligible records indexed elsewhere. Cross-database author names and citation metadata are not fully equivalent, so co-authorship interpretation was kept descriptive, and database-specific citation metrics were not directly pooled. Second, the biological evidence base remains small and heterogeneous with respect to study design, human biospecimen versus cell model, cadmium exposure metric, dose and duration, assay platform, RNA normalization, sample size, and statistical thresholds. Third, directionality is based on study-level reporting rather than standardized effect sizes; the analysis therefore cannot estimate a pooled magnitude of miRNA change. Fourth, some publications report family-level or precursor-level miRNA names, which can obscure mature-arm specificity. Fifth, the existing target-network component is exploratory and potentially sensitive to target-curation density; cadmium-specific hub or pathway claims require unbiased target collection, formal enrichment, and appropriate background or null-model comparisons. Sixth, publication and selective-reporting bias cannot be excluded. The directionality synthesis is based on a small number of published studies per recurrent miRNA, and the available study counts and heterogeneous designs precluded a meaningful formal assessment of publication bias; null or non-significant miRNA findings may therefore be underrepresented.

Future research should prioritize prospective, adequately powered, multicenter human studies with repeated measurements of cadmium exposure and miRNA expression. Standardized reporting should include the mature miRNA arm, assay platform, normalization strategy, sample processing, detection limits, exposure matrix, timing, dose, and key confounders. Matched miRNA-seq, mRNA-seq, proteomic, metabolomic, and epigenomic measurements would permit directionally coherent miRNA-mRNA integration and distinguish predicted regulation from demonstrated target suppression. Studies should stratify or adjust for smoking, diet, renal function, age, sex, occupational co-exposures, and inflammatory diseases. Beyond discovery-level validation, translating any candidate miRNA signature into a population-based screening tool would face further practical barriers that are largely unaddressed in the current literature: assay standardization across laboratories and platforms (no consensus reference gene or spike-in control has been adopted across the cadmium–miRNA literature reviewed here); inter-individual variability in baseline miRNA expression, for which reference ranges in unexposed populations are rarely reported; and cost-effectiveness relative to established, cheaper exposure biomarkers such as blood or urinary cadmium. Candidate panels emerging from discovery studies should be tested in external cohorts, and causal mediation or perturbation experiments should evaluate whether modulating prioritized axes—particularly miR-146a/*b-IRAK1*/*TRAF6* [51], miR-221/222-*PTEN/TIMP3/CDKN1B* [49], miR-126-*PIK3R2/SPRED1* [50], and miR-30-*BECN1/ATG5* [52]—alters cadmium-induced phenotypes. Similar priorities for standardized exposure characterization and miRNA reporting have been emphasized in recent reviews of environmental miRNA responses [24,29,33].

Overall, the integrated evidence supports a model in which cadmium exposure reshapes miRNA regulation across interconnected inflammatory, oxidative, survival, endothelial, autophagic, and tissue-remodeling pathways. The most informative signals are not simply the most frequently reported miRNAs, but those that combine recurrence, directional consistency, biologically coherent targets, and validation in relevant human systems. This framework provides a rational basis for selecting miRNA panels and target pathways for mechanistic and translational studies of cadmium toxicity.

## 4. Materials and Methods

### 4.1. Bibliometric Design and Data Source

This study employed a bibliometric approach to evaluate the development, thematic structure, and collaboration patterns of research on cadmium exposure and microRNAs. To improve literature coverage and reduce database-specific indexing bias, three complementary bibliographic databases were used: PubMed/MEDLINE, Scopus, and Web of Science Core Collection. To avoid mixing distinct units of analysis, two analytically separate record sets were defined. The bibliometric corpus comprised eligible original articles and reviews with a substantive cadmium–microRNA relationship and was used for publication, keyword, citation, and collaboration analyses. A separate biological evidence corpus was restricted to primary studies involving human participants, human biospecimens, or human-derived experimental models and was used only for the downstream miRNA directionality and bioinformatics analyses.

### 4.2. Search Strategy and Data Preparation

The literature search was conducted in PubMed/MEDLINE, Scopus, and Web of Science Core Collection on 13 August 2026. A common conceptual search strategy combined cadmium-related terms (“cadmium”, “cadmium exposure”, “cadmium toxicity”, “cadmium poisoning”, and “cadmium chloride”) with microRNA-related terms (“microRNA”, “miRNA”, “miRNAs”, and “miR-”). Database-specific field syntax was adapted to the indexing structure of each platform while preserving the same conceptual scope. Searches were restricted to English-language journal articles and reviews where the relevant database filters permitted equivalent restrictions.

The searches retrieved 319 records from PubMed/MEDLINE, 692 from Scopus, and 500 from Web of Science, yielding 1511 source records before harmonization. Eleven non-English records and 26 records with ineligible Web of Science document types were removed before deduplication, leaving 1474 source records. Cross-database deduplication was then performed sequentially using DOI, PMID, and normalized exact-title matching. This procedure removed 657 duplicate source records and yielded 817 unique records for title/abstract screening.

For the bibliometric corpus, titles and abstracts were screened for a substantive cadmium–microRNA or cadmium–small-RNA relationship. Records in which cadmium and microRNA terminology appeared only incidentally, or in which cadmium-containing materials were used solely as analytical or sensing components without a biological cadmium–microRNA relationship, were excluded. After screening, 286 publications were retained in the final bibliometric corpus. Fourteen eligible publications were identified in PubMed and/or Web of Science but were absent from the Scopus dataset, demonstrating the additional coverage gained by the multi-database strategy.

Before bibliometric analysis, metadata were harmonized across databases. Spelling variants, singular and plural forms, capitalization variants, and synonymous author-keyword terms representing the same concept were standardized using a predefined VOSviewer (version 1.6.18) thesaurus. Examples included consolidation of “miRNA”, “miRNAs”, and “microRNA” under “microRNA”, normalization of circRNA and long-noncoding-RNA variants, and harmonization of heavy-metal terminology. Conceptually distinct terms were retained separately.

### 4.3. Bibliometric and Network Analyses

VOSviewer (version 1.6.18, Centre for Science and Technology Studies, Leiden University, Leiden, The Netherlands) was used for author-keyword co-occurrence mapping of the final 286-record bibliometric corpus. VOSviewer was retained specifically for this keyword-network analysis because it is the field-standard tool for keyword co-occurrence mapping and provides built-in thesaurus-based term harmonization and association-strength normalization that are widely used and directly comparable across the bibliometric literature; by contrast, the co-authorship network (Section 2.2) required bespoke handling of merged, cross-database author-name exports and was therefore constructed using a custom, fully scripted Python pipeline (Section 4.3) to ensure exact reproducibility of author retention, deduplication, and clustering decisions. Because index-keyword systems differ across PubMed/MEDLINE, Scopus, and Web of Science, author keywords were selected as the primary unit for cross-database keyword analysis. The network, temporal overlay, and density panels in Figure 1 were generated in VOSviewer from the final harmonized author-keyword data using full counting and association-strength normalization. Node size represents keyword occurrence, link thickness represents co-occurrence strength, spatial proximity represents relatedness, and overlay color represents mean publication year.

The primary keyword analysis required a minimum author-keyword occurrence of three. VOSviewer clustering was performed at the standard resolution parameter of 1.00, and the resulting clusters were interpreted descriptively from their dominant terms. A second analysis using a minimum occurrence of five was prespecified as a sensitivity analysis to assess whether the principal thematic structure remained stable after removal of lower-frequency terms. Network, overlay, and density visualizations were examined for the primary threshold, and the threshold-five network was used as a robustness check. The source exports, final bibliometric corpus, VOSviewer thesaurus, and threshold map/network files are supplied in the accompanying submission reproducibility materials (Data Availability Statement).

Co-authorship analysis was reproduced in Python 3.13.5 (Python Software Foundation, Wilmington, DE, USA) using pandas 2.2.3 and NetworkX 3.6.1 [53]. Author strings were split on semicolons and treated as bibliographic author-name labels; exact duplicate labels within a publication were collapsed, but no ORCID-based person disambiguation was imposed. Of 1234 unique exported author labels, 283 occurred in at least two documents and entered the eligible weighted graph. Edge weight was the number of publications co-authored by a pair of labels, and total link strength (TLS) was defined as the weighted degree in this full eligible graph. The 26 labels with the highest full-graph TLS were selected for Figure 2; their induced subgraph contained 119 links with a summed edge weight of 292. Collaboration clusters were obtained using NetworkX Louvain community detection (resolution = 1.00, seed = 42), yielding three clusters of 15, 9, and 2 author-name nodes. The complete reconstruction script and top-26 node/edge tables are supplied in the accompanying reproducibility package. Database-specific citation indicators were interpreted only within their source database. Field-weighted citation impact (FWCI) was used as a normalized citation indicator comparing a publication’s citation impact with that expected for similar publications by subject field, year, and publication type [54]. Microsoft Excel (Microsoft 365; Microsoft Corporation, Redmond, WA, USA) was used for data organization and descriptive tabulation.

### 4.4. Bioinformatics and Network Analysis

The bioinformatics component was designed as a hypothesis-generating extension of the bibliometric synthesis. Because the included publications did not provide a harmonized raw expression matrix, the analyses were performed at the study-summary level and addressed three complementary questions: (i) whether recurrent miRNAs showed a consistent direction of change across studies; (ii) which experimentally supported target genes were shared among candidate miRNAs included in the exploratory mechanism-oriented network; and (iii) which cadmium-relevant biological processes were represented by these targets.

### 4.5. Selection and Nomenclature Normalization of Recurrent miRNAs

All miRNAs reported in at least three included studies were entered into the direction-consistency analysis. miRNA names were standardized according to mature human miRNA nomenclature and current miRBase guidance [55]. When the original publication reported only a precursor or family-level designation or did not specify the 5p/3p arm, the reported family designation was retained rather than assigning an unsupported mature species. The exploratory target-network analysis was treated as a separate mechanistic component rather than as an extension of the recurrence vote count. Candidate nodes were included when the human-relevant literature supported sufficiently consistent arm-specific nomenclature and a conservative set of experimentally validated human targets: hsa-miR-146a-5p, hsa-miR-146b-5p, hsa-miR-21-5p, hsa-miR-155-5p, hsa-miR-126-3p, hsa-miR-221-3p, hsa-miR-222-3p, hsa-miR-26a-5p, hsa-miR-26b-5p, and the miR-30 family (5p). Network inclusion therefore does not imply that each node met the final ≥3-study recurrence threshold.

### 4.6. Cross-Study Direction-Consistency Analysis

For each recurrent miRNA, the numbers of studies reporting increased expression (n_up), decreased expression (n_down), or no significant change (n_unchanged) were extracted from Table 1. A directionality index was calculated as DI = (n_up − n_down)/n_total. DI ranges from −1, indicating a uniformly decreased pattern, to +1, indicating a uniformly increased pattern. The dominant-direction proportion was calculated as the largest of n_up, n_down, or n_unchanged divided by n_total. To quantify heterogeneity across the three possible directions, normalized Shannon entropy was calculated as H = −sum[p_i ln(p_i)]/ln(3), where p_i represents the proportion of studies in each direction category. H approaches 0 when all studies show the same direction and approaches 1 when the three directions are evenly distributed. Because only study-level counts were available, pooled effect sizes and formal meta-analytic models were not performed. However, to test whether the dominant-direction proportion for each recurrent miRNA departed from a 50:50 null expectation, we computed an exact two-sided binomial test together with an exact (Clopper–Pearson) 95% confidence interval for the dominant-direction proportion, using SciPy 1.14 (scipy.stats.binomtest; scipy.stats.beta) in Python 3.13.5. As a robustness check against pseudo-replication, we additionally collapsed studies sharing the same research group and model system (Research_Group_Model_ID) into a single evidence unit and recomputed all counts; this sensitivity analysis is reported alongside the primary results in Section 2.5. To assess whether the observed directional heterogeneity might reflect technical rather than biological differences, we additionally extracted, for each of the ten studies contributing to the five recurrent miRNAs, the exposure matrix/model system, quantification platform, cadmium dose or exposure level, and normalization strategy, from the primary publication’s abstract and accessible full text (Table 4). Where a detail could not be confirmed from openly accessible text, it is reported as such rather than inferred or omitted.

### 4.7. Target-Gene Curation and Network Construction

A conservative, mechanism-oriented miRNA–target set was assembled using experimentally supported human miRNA–target interactions catalogued in miRTarBase 2025 [56] and the primary mechanistic literature represented within the included studies. Priority was given to interactions supported by reporter assays, Western blotting, quantitative PCR, or gain- and loss-of-function experiments in human cells or tissues. TargetScanHuman release 8.0 was used as orthogonal support for canonical seed–family relationships and target plausibility; predicted-only interactions were not counted as network edges [57,58]. Interactions were retained when the target gene was relevant to at least one prespecified cadmium-related process: oxidative-stress regulation, innate immune/TLR-NF-κB signaling, apoptosis and cell-cycle control, autophagy, PI3K-AKT and growth-factor signaling, TGF-beta/epithelial–mesenchymal transition (EMT), extracellular-matrix remodeling and fibrosis, endothelial/angiogenic regulation, or epigenetic regulation and DNA repair. Gene aliases were harmonized to current human gene symbols, and duplicate interactions were removed.

The final bipartite network contained miRNA nodes, target-gene nodes, and experimentally supported miRNA–target edges. Node degree was defined as the number of direct connections. Genes targeted by at least two candidate miRNAs in this exploratory network were considered shared-target hubs. For each pair of miRNAs, target overlap was summarized using the Jaccard coefficient, calculated as the number of shared targets divided by the total number of unique targets in the two target sets. Network calculations and visualizations were performed in Python 3.13.5 using pandas 2.2.3, NetworkX 3.6.1, and Matplotlib 3.10.8 [53]. Functional-module annotation was descriptive and knowledge-guided; formal over-representation *p*-values were not calculated because the intentionally restricted target set was not an exhaustive genome-wide target universe.

To evaluate whether the observed hub status of *PTEN* and the elevated pairwise target overlap among seed–family miRNA pairs (hsa-miR-221-3p/hsa-miR-222-3p, hsa-miR-146a-5p/hsa-miR-146b-5p, and hsa-miR-26a-5p/hsa-miR-26b-5p) could be explained by chance, we implemented a random-reassignment null model in Python (NumPy 2.1). For each of 20,000 iterations, every miRNA’s observed number of curated targets was held fixed while its specific target genes were redrawn without replacement, uniformly at random, from the same 51-gene curated pool used in the primary analysis, independently for each miRNA. We recorded, for each iteration, the network’s maximum gene degree, PTEN’s degree specifically, and the Jaccard coefficient for each of the three seed–family pairs and have reported the corresponding empirical *p*-values (proportion of null iterations meeting or exceeding the observed value) in Section 2.6. We additionally computed the Jaccard coefficient for all 42 possible cross-family (non-seed-sharing) miRNA pairs in the observed network for direct comparison with the three within-family pairs. This null model is explicitly conditioned on the same restricted, mechanism-oriented target set used in the primary analysis (Table 5) rather than a genome-wide or miRTarBase-wide background, and its results should therefore be interpreted as testing whether the observed pattern is nontrivial given the curated set, not as a claim of genome-wide statistical enrichment.

### 4.8. Study Selection, Eligibility Criteria, and Analytical Record Sets

Study selection was organized in two stages to distinguish bibliometric mapping from biological evidence synthesis. First, the three-database search yielded 1511 source records; after language/document-type filtering and cross-database deduplication, 817 unique records underwent title/abstract screening. A total of 286 publications with a substantive cadmium–microRNA relationship were retained in the bibliometric corpus and used for bibliometric mapping. Second, primary human-relevant studies were evaluated separately for the biological evidence corpus. This second corpus was restricted to studies involving human participants, human biospecimens, or human-derived experimental models in which cadmium-associated miRNA evidence could be directly assessed. Reviews were eligible for bibliometric mapping but were not counted as biological observations or directional votes. Animal-only studies, non-human cell models, editorials, conference papers, and studies without an evaluable cadmium–miRNA relationship were excluded from the biological evidence corpus. Of the 56 human-relevant candidate records assessed for full-text eligibility, 32 studies provided direct, extractable human cadmium–miRNA expression evidence and were retained in the biological evidence corpus; 24 were excluded from directionality analyses because they provided mechanistic context only without a clean human-exposed-versus-control direction (*n* = 12), association-only or mixed-exposure evidence unsuitable for directional vote counting (*n* = 10), or a secondary in silico reanalysis of an already represented dataset (*n* = 2). Thirty-one of the 32 retained studies provided source-verified named-miRNA directions. GSE246130 was retained in the evidence inventory for completeness but excluded from named-miRNA recurrence and directionality analyses because a complete arm-resolved differential-expression list was unavailable from the accessible source material.

To provide a transparent, if necessarily simplified, indicator of methodological completeness across the biological evidence corpus, each of the 32 studies was scored on four prespecified reporting criteria: whether the cadmium dose/exposure regimen, the quantification platform, the normalization strategy, and the sample size/replicate structure were explicitly stated in the accessible text (1 point each; maximum 4). This is a completeness/transparency indicator rather than a formal risk-of-bias instrument such as RoB2 or ROBINS-I, which are designed for randomized or comparative epidemiological designs and do not map cleanly onto this heterogeneous mixture of in vitro mechanistic and human biomonitoring studies; no study was excluded on the basis of this score. Full per-study scores, together with a completed PRISMA 2020 reporting/applicability checklist, are provided in Supplementary Table S1 and Supplementary File S1, respectively. This study was not prospectively registered in PROSPERO or an equivalent registry.

**Figure 6:**
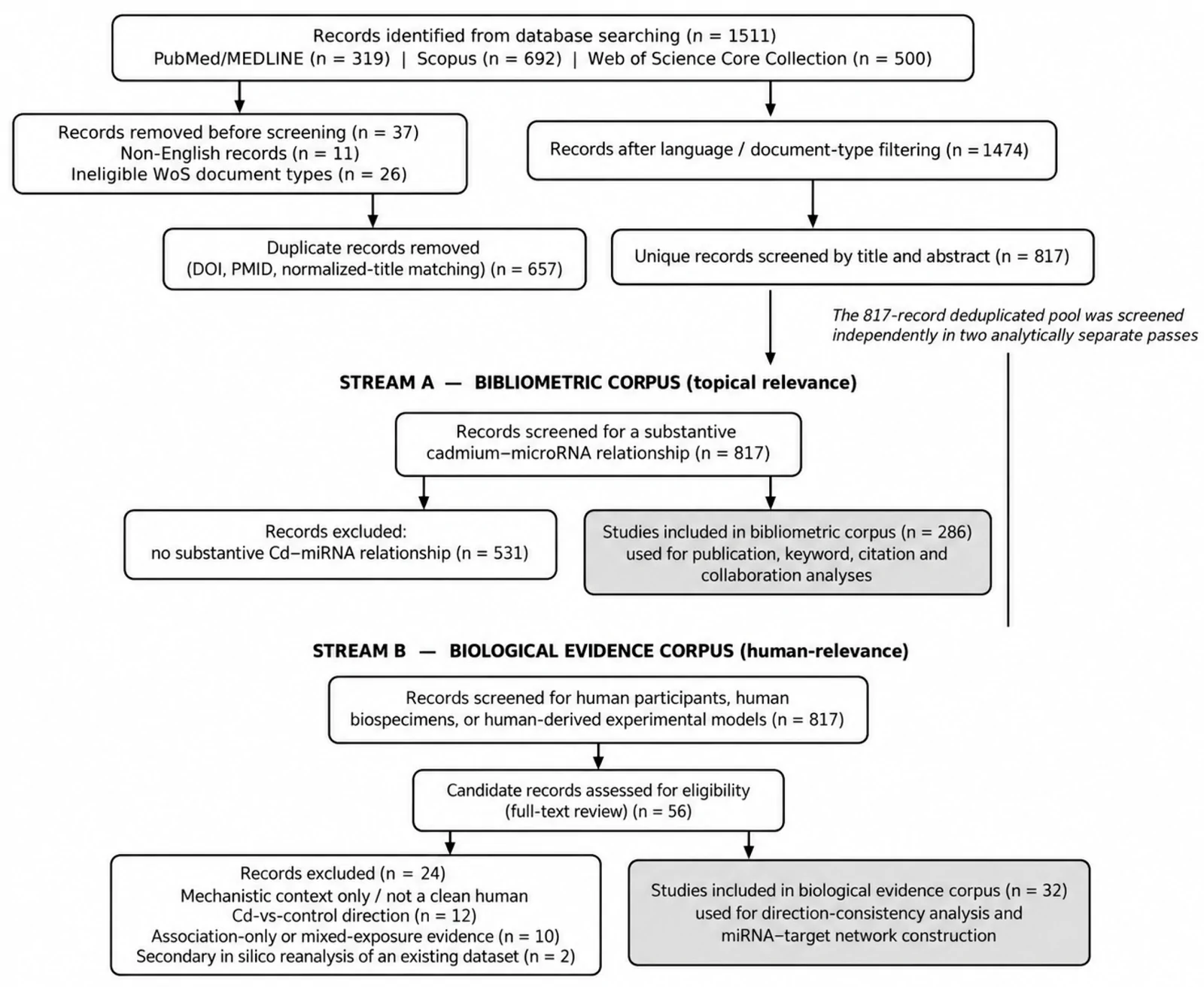
PRISMA-style identification and selection flow diagram. Stream A represents the bibliometric corpus (*n* = 286) and Stream B the biological evidence corpus (*n* = 32). Thirty-one studies contributed source-verified named-miRNA evidence; GSE246130 was retained for transparency/completeness assessment but excluded from named-miRNA recurrence and directionality analyses because a complete arm-resolved differential-expression list was unavailable in the finalized evidence extraction. Arrows indicate sequential progression through identification, screening, eligibility assessment, and final corpus assignment.

The overall study-selection process and the two analytically distinct evidence streams are summarized in Figure 6.

## 5. Conclusions

This synthesis shows that a multi-database search provides a broader view of the cadmium–miRNA literature than a Scopus-only approach. The multi-database strategy yielded 817 unique records after deduplication and a final bibliometric corpus of 286 topic-relevant publications, with a stable thematic core centered on cadmium, microRNA, heavy-metal toxicity, apoptosis, and cadmium-stress responses. The stricter biological synthesis, restricted to the 32 studies retained in the biological evidence corpus, however, provides a more cautious picture of recurrent miRNA directionality: miR-155 showed a balanced up/down pattern, while the let-7 and miR-30 families and miR-146b and miR-222 showed only modest directional tendencies based on small numbers of studies.

These findings do not support labeling any recurrent miRNA as a uniformly reproducible cadmium marker at present. Mechanistic target relationships remain useful for hypothesis generation, but shared hubs and pathway annotations should not be interpreted as cadmium-specific until evaluated against unbiased target backgrounds and formal enrichment or null models. The strongest contribution of this analysis is therefore the combination of broader literature coverage, explicit separation of bibliometric and biological evidence sets, and a more conservative interpretation of cross-study miRNA signals.

Future translational work should prioritize prospective and adequately powered human studies with standardized cadmium exposure assessment, mature-miRNA nomenclature, pre-analytical handling, assay platforms, normalization procedures, and reporting of effect sizes with uncertainty. External validation in independent populations and integration with matched mRNA, protein, and pathway-level data will be necessary before cadmium-associated miRNA signatures can be considered for occupational or population-based biomarker applications.

## Figures and Tables

**Table 1:** Most frequently reported miRNAs associated with cadmium exposure in the final biological evidence corpus.

miRNA	↑ (Increase)	↓ (Decrease)	Total
miR-155	2	2	4
let-7 family	1	2	3
miR-146b	2	1	3
miR-222	2	1	3
miR-30 family	1	2	3

**Table 2:** The five most highly cited publications on cadmium and miRNAs (FWCI = field-weighted citation impact; citation counts and FWCI values reflect the Scopus export snapshot of 13 August 2026 and may change over time).

Article Title	Author(s)	Citations	FWCI	miRNA
LncRNA OIP5-AS1 inhibits ferroptosis in prostate cancer with long-term cadmium exposure through miR-128-3p/SLC7A11 signaling	Zhang Y. et al. (2021) [35]	167	9.15	miR-128-3p
MiR-101 and miR-144 Regulate the Expression of the CFTR Chloride Channel in the Lung	Hassan F. et al. (2012) [37]	140	2.98	miR-101, miR-144
Cadmium and its epigenetic effects	Wang B. et al. (2012) [38]	138	3.10	Multiple
LncRNA MT1DP aggravates cadmium-induced oxidative stress by repressing Nrf2 via miR-365	Gao M. et al. (2018) [39]	71	2.94	miR-365
Whole genome analysis and microRNAs regulation in HepG2 cells exposed to cadmium	Fabbri M. et al. (2012) [40]	67	3.69	let-7 family

**Table 3:** Direction-consistency metrics for recurrent cadmium-associated miRNAs.

miRNA	Up/Down/ No Change	*n*	DI	Dominant Proportion	H	Exact 95% CI (2-Sided *p*)	Interpretation
miR-155	2/2/0	4	0.00	0.50	0.631	6.8–93.2% (*p* = 1.00)	Balanced up/down pattern; marked heterogeneity
let-7 family	1/2/0	3	−0.33	0.67	0.579	9.4–99.2% (*p* = 1.00)	Heterogeneous; downward tendency
miR-146b	2/1/0	3	+0.33	0.67	0.579	9.4–99.2% (*p* = 1.00)	Heterogeneous; upward tendency
miR-222	2/1/0	3	+0.33	0.67	0.579	9.4–99.2% (*p* = 1.00)	Heterogeneous; upward tendency
miR-30 family	1/2/0	3	−0.33	0.67	0.579	9.4–99.2% (*p* = 1.00)	Heterogeneous; downward tendency

**Table 4:** Exposure matrix, model system, platform, dose/exposure, and normalization reported for the ten studies contributing to the five recurrent miRNAs.

Study	Exposure Matrix/Model	Platform	Dose/Exposure	Normalizer
Fabbri et al., 2012 [40]	HepG2 human hepatoma cells	TaqMan array microRNA cards (low-density array)	10 µM CdCl2, 24 h	U6
Ngalame et al., 2016 [42]	RWPE-1 → CTPE (chronically Cd-transformed prostate epithelium)	qRT-PCR cancer-miRNA array + selected qPCR	10 µM Cd, continuous 8 weeks (transformation model)	SNORD61/68/72/95/96A + RNU6B/RNU6-2 (array); RNU6-2 (qPCR)
Lemaire et al., 2020 [43]	RPTEC/hTERT; HK-2 validation	QuantStudio 12K Flex TaqMan OpenArray + qPCR validation	RPTEC/hTERT: 10 µM CdCl2, 24 h; HK-2: 20 µM, 24 h	U6 snRNA
Xiong et al., 2022 [47]	Human placental trophoblasts	miRNA microarray + qPCR	Cd-treated trophoblasts; exact in vitro dose/time not confirmed from accessible primary text	Not confirmed in accessible primary text
Brooks et al., 2016 [44]	JEG-3 human placental trophoblast cells	Agilent Human miRNA v16 (8 × 60 K) microarray + targeted qRT-PCR	25 µM CdCl2, 48 h (array); 1 and 10 µM, 48 h (targeted qPCR)	U6
Mortoglou et al., 2022 [45]	PANC-1 and MiaPaCa-2 human pancreatic ductal adenocarcinoma cells	miRCURY LNA RT + SYBR Green qRT-PCR	50 µM CdCl2, 14 days	U6-snRNA
Mo et al., 2026 [48]	Primary human bone-marrow mesenchymal stem cells	Mechanistic miRNA analysis; full platform details not confirmed from accessible primary text	0, 0.46, and 0.92 mg/L CdCl2	Not confirmed in accessible primary text
Mitra et al., 2023 [28]	Human serum/blood; occupationally exposed workers (*n* = 100) vs. controls (*n* = 80)	Real-time PCR	Chronic occupational exposure; blood Cd 2.40 vs. 0.90 µg/L	Not specified in accessible primary text
Tanwar et al., 2019 [46]	BEAS-2B and BEP2D human lung epithelial cells	qScript miRNA cDNA + SYBR qPCR (7900HT); transcriptomic screening	0, 2.5, 5, and 10 µM CdCl2, 72 h	SNORD44
Zheng et al., 2021 [41]	Human COPD case-control cohort plus BEAS-2B validation	Serum miR-30 RT-PCR; Cd measured by atomic absorption	Circulatory Cd exposure; complementary BEAS-2B Cd experiments	Not confirmed in accessible primary text

**Table 5:** Curated mechanism-oriented target sets used for network construction.

miRNA	Curated Target Genes	Principal Biological Context
hsa-miR-146a-5p	*IRAK1; TRAF6; STAT1; TLR4; NOTCH1*	Innate immune/TLR-NF-κB; apoptosis
hsa-miR-146b-5p	*IRAK1; TRAF6; STAT1; SMAD4; MMP16*	Innate immune signaling; TGF-beta/EMT; matrix remodeling
hsa-miR-21-5p	*PTEN; PDCD4; SPRY1; SPRY2; RECK; TIMP3; TGFBR2; SMAD7; MSH2*	PI3K-AKT; apoptosis; TGF-beta/EMT; matrix remodeling; DNA repair
hsa-miR-155-5p	*SOCS1; INPP5D; BACH1; FOXO3; MYD88; TAB2; SPI1; CEBPB; RIPK1; IKBKE*	Immune/inflammatory signaling; oxidative stress; apoptosis
miR-30 family (5p)	*BECN1; ATG5; DNMT3A; SNAI1; VIM; ITGB3; RUNX2*	Autophagy; epigenetic regulation; EMT; matrix/cytoskeletal regulation
hsa-miR-26a-5p	*PTEN; EZH2; SMAD1; SMAD4; CCND2; HMGA2*	PI3K-AKT; epigenetic regulation; TGF-beta; cell cycle
hsa-miR-26b-5p	*PTEN; EPHA2; PTGS2; CCND2; EZH2*	PI3K-AKT; oxidative/inflammatory response; cell cycle; epigenetic regulation
hsa-miR-221-3p	*CDKN1B; CDKN1C; PTEN; TIMP3; BMF; BBC3*	Cell-cycle control; PI3K-AKT; matrix remodeling; apoptosis
hsa-miR-126-3p	*PIK3R2; SPRED1; VCAM1; IRS1; CRK; EGFL7*	Endothelial/angiogenic regulation; PI3K-AKT; inflammation
hsa-miR-222-3p	*CDKN1B; CDKN1C; PTEN; TIMP3; ETS1; STAT5A; BBC3*	Cell-cycle control; PI3K-AKT; matrix remodeling; apoptosis; transcriptional regulation

**Table 6:** Functional modules represented in the exploratory curated candidate-miRNA–target network.

Functional Module	Genes, *n*	miRNAs, *n*	Representative Genes
Innate immune/TLR-NF-κB signaling	13	4	*CEBPB; IKBKE; INPP5D; IRAK1; MYD88; RIPK1; SOCS1; SPI1; STAT1; TAB2; TLR4; TRAF6; VCAM1*
TGF-beta/EMT, matrix remodeling, and fibrosis	10	6	*ITGB3; MMP16; RECK; SMAD1; SMAD4; SMAD7; SNAI1; TGFBR2; TIMP3; VIM*
PI3K-AKT and growth-factor signaling	8	6	*CRK; EGFL7; EPHA2; IRS1; PIK3R2; PTEN; SPRY1; SPRY2*
Apoptosis and cell-cycle control	8	7	*BBC3; BMF; CCND2; CDKN1B; CDKN1C; FOXO3; NOTCH1; PDCD4*
Endothelial/angiogenic regulation	7	3	*CRK; EGFL7; EPHA2; ETS1; PIK3R2; SPRED1; VCAM1*
Oxidative-stress response	4	6	*BACH1; FOXO3; PTEN; PTGS2*
Epigenetic regulation and DNA repair	3	4	*DNMT3A; EZH2; MSH2*
Autophagy	2	1	*ATG5; BECN1*

## Data Availability

A submission reproducibility package accompanies this manuscript. It includes the PubMed/MEDLINE, Scopus, and Web of Science source exports used for the 13 August 2026 search; the final 286-record bibliometric corpus; the keyword-thesaurus mapping and VOSviewer ≥3/≥5 threshold files; a Python co-authorship reconstruction script with top-26 node/edge tables reproducing the Figure 2 network counts and clusters; the 32-study methodological-completeness table plus a CSV mirror of that final evidence inventory; a 16-row study-level recurrent-miRNA vote table (10 unique contributing studies) with a Python script reproducing the Table 3 study counts, exact binomial and Clopper–Pearson statistics, directionality index, entropy, research-group/model collapse sensitivity, and ≥4-study threshold check; and the curated miRNA–target edge list with a Python script reproducing the 20,000-iteration network null-model analyses. Supplementary File S1 contains the PRISMA 2020 reporting/applicability checklist. No public repository DOI or URL has been assigned; these materials are supplied directly with the submission.

## Supplementary Materials

The following supporting information can be downloaded at: https://www.mdpi.com/article/10.3390/ijms27188051/s1. References [25,28,36,37,39,40,41,42,43,44,45,46,47,48,59,60,61,62,63,64,65,66,67,68,69,70,71,72,73,74,75,76] are cited in Table S1.
