# Supplementary material for: Mapping the MicroRNA Landscape of Cadmium Exposure: A Bibliometric and Bioinformatics Analysis of Molecular Targets and Regulatory Pathways
Source: Int J Mol Sci. 2026 Sep 10;27(18):8051. doi: 10.3390/ijms27188051 (PMC13607735; doi:10.3390/ijms27188051)
Supplement: Supplementary file 1 [file ijms-27-08051-s001.zip › Supplementary_File_S1.pdf]

PRISMA 2020 Reporting Checklist — Applicability to IJMS-4474757

Mapping the MicroRNA Landscape of Cadmium Exposure: A Bibliometric and Bioinformatics Analysis of Molecular Targets and Regulatory Pathways

Scope note. This author-completed checklist transparently reports how PRISMA 2020 items apply to this hybrid bibliometric and structured biological-evidence synthesis. It does not reclassify the article as a conventional systematic review. Items that are not applicable or not fully met are identified as such rather than inferred. Reference numbers cited in this checklist correspond to the main manuscript reference list.

Item	PRISMA 2020 topic (paraphrased)	Status	Manuscript location / note
1	Identification in title	Partial	The title identifies bibliometric and bioinformatics analyses but does not label the article a systematic review.
2	Structured summary of objectives, sources, methods, results, and conclusions	Partial	Abstract: databases, record counts, principal analyses, results, limitations, and conclusion are reported in a single-paragraph journal format.
3	Rationale in context of existing knowledge	Yes	Introduction, especially Section 1.3 and comparison with Koushki et al. [29], Wallace et al. [33], and Chaiwangyen et al. [24].
4	Explicit objectives/questions	Yes	End of Introduction (Section 1.3) and Section 4.4.
5	Eligibility criteria	Yes	Sections 4.1, 4.2, and 4.8; bibliometric and biological-evidence corpora are defined separately.
6	Information sources and last search date	Yes	Section 4.2: PubMed/MEDLINE, Scopus, and Web of Science Core Collection; searched 13 August 2026.
7	Search strategy sufficiently documented	Partial	Section 4.2 gives the conceptual strategy and database-specific adaptation. Source exports and thesaurus are supplied in the reproducibility package.
8	Study-selection process	Yes	Section 4.8 and Figure 6.
9	Data-collection/extraction process	Partial	Sections 4.5–4.8 describe study-level extraction and technical-data verification; no independent duplicate-extraction form is reported for every variable.
10	Data items and definitions	Yes	Sections 4.5–4.8 define miRNA nomenclature, direction counts, technical variables, target curation, and completeness criteria.
11	Risk-of-bias assessment	Partial	A four-criterion methodological-completeness/transparency indicator is reported (Supplementary Table S1); it is explicitly not RoB2/ROBINS-I.
12	Effect measures	Partial	No pooled effect-size meta-analysis was feasible. Directionality index, dominant-direction proportion, normalized entropy, exact CI, and exact binomial p values are defined in Section 4.6.
13	Synthesis methods	Yes	Sections 4.3–4.7: bibliometric mapping, direction-consistency analysis, sensitivity analysis, curated network, Jaccard analysis, and null model.
14	Assessment of reporting bias due to missing results	No	No formal reporting-bias analysis was performed; publication/selective-reporting bias is explicitly acknowledged in the Discussion as an unresolved limitation.
15	Certainty/confidence assessment	No	No GRADE-style certainty assessment was performed.
16	Study-selection results	Yes	Figure 6 and Section 4.8 report source records, deduplication, screening, corpus sizes, and exclusion categories.
17	Characteristics of included studies	Yes	Supplementary Table S1 (including the Ref. column linking all 32 studies to the main manuscript reference list) and Table 4 summarize study/model and technical characteristics; the reproducibility package contains the final bibliometric corpus.
18	Risk-of-bias/completeness results	Partial	Supplementary Table S1 reports the methodological-completeness indicator for all 32 biological-evidence studies and links each study to its corresponding main-manuscript reference number.
19	Results of individual studies	Partial	Study-level directionality is synthesized for recurrent miRNAs; not all individual effect sizes were available or pooled.
20	Results of syntheses	Yes	Results 2.1–2.7, Tables 1–6, and Figures 1–6.
21	Reporting-bias results	No	No formal reporting-bias assessment was performed; this limitation is explicitly discussed in the manuscript.

22	Certainty of evidence results	No	No formal certainty-of-evidence grading was performed.
23	Discussion: interpretation, limitations, implications	Yes	Discussion Section 3 includes interpretation, technical/biological heterogeneity, limitations, and future research priorities.
24	Registration/protocol	No	Section 4.8 explicitly states that the study was not prospectively registered in PROSPERO or an equivalent registry.
25	Support/funding	Yes	Funding statement: no external funding.
26	Competing interests	Yes	Conflicts of Interest statement: authors declare no conflicts.
27	Availability of data, materials, and code	Partial	Submission reproducibility package supplies raw database exports, final bibliometric corpus, thesaurus, methodological-completeness audit, and scripts for the principal directionality and network-null analyses. No public repository DOI/URL is claimed.

Status definitions: Yes = substantially reported; Partial = partly applicable/reported with explicit limitation; No = not performed/reported; N/A = not applicable.

Final synchronization note: This checklist was updated against the final proof-synchronized manuscript and supporting materials after primary-source verification of technical study characteristics. Supplementary Table S1 now includes a Ref. column linking each of the 32 biological-evidence studies to the main manuscript reference list, and the Supplementary Materials section of the main manuscript cites the corresponding reference set.
